# Projection of key meteorological hazard factors in Xiongan new area of Hebei Province, China

**DOI:** 10.1038/s41598-021-98160-z

**Published:** 2021-09-21

**Authors:** Dapeng Huang, Yaoming Liao, Zhenyu Han

**Affiliations:** 1 National Climate Center, China Meteorological Administration, Beijing, 100081 China; 2 Collaborative Innovation Center on Forecast and Evaluation of Meteorological Disasters, Nanjing University of Information Science and Technology, Nanjing, 210044 China

**Keywords:** Climate sciences, Natural hazards

## Abstract

It is of great importance to explore the future spatiotemporal dynamics of key meteorological hazard factors in Xiongan New Area, an area of great strategic significance under construction in China. Based on 6.25 km high-resolution downscaling projection data under RCP4.5 and RCP8.5 scenarios, Mann–Kendall trend and linear trend were analyzed, and then stationary generalized extreme value (GEV) and time-varying GEV methods were determined to calculate the extremes of four key meteorological hazard factors with return periods of 10, 20, 30, 50 and 100 years during the projection period 1991–2050. Results show that extremes of annual maximum daily precipitation and annual maximum amount of consecutive precipitation under two climate scenarios will not increase too much. Extremes of annual maximum temperature will increase by above 1.5 °C under RCP4.5 scenario in most grids and above 1.9 °C under RCP8.5 scenario. Extremes of annual longest consecutive high-temperature days will increase by above 0.9d under RCP4.5 scenario and above 1.6d under RCP8.5 scenario. On the whole, the hazard of flood disaster will hardly show any change up to 2050, but there will be relatively higher flood hazard in Xiongxian county and its adjacent region. All regions in Xiongan New Area will face high hazard of high-temperature disaster.

## Introduction

On April 1st, 2017, China issued a document deciding to establish Xiongan New Area^1^. The creation of Xiongan New Area is “a strategy that will have lasting importance for the millennium to come, and a significant national event”. The national strategic decision to establish Xiongan New Area has attracted great attention from the academic community. Scholars have carried out a lot of researches on Xiongan New Area from the perspectives of multiple disciplines, which provides important scientific references for the construction of Xiongan New Area^2–12^. However, the historical and future spatiotemporal characteristics of key meteorological hazard factors in Xiongan New Area have not been studied deeply. In the past 700 years, the severe floods in Xiongxian county of Xiongan New Area averaged once every 12 years. In August 1963, the water level of Baiyangdian Lake exceeded 11 m a.s.l for 13 days^5^. The master plan document for Xiongan New Area published in April 2018 clearly proposed the construction of a flood prevention security system to ensure the New Area's safety and flood prevention. Therefore, the flood disaster risk in Xiongan New Area, especially during the major construction phase deserves more attention. Previous studies on flood disaster in Xiongan New Area mainly focused on the analysis and evaluation of historical disasters^13^, without giving enough attention to the spatiotemporal characteristics and future changes of the hazard factors of meteorological disaster. The research on the comprehensive risk zoning of high-temperature disasters in the Beijing-Tianjin-Hebei region shows that Rongcheng, Anxin, and Xiongxian counties of Xiongan New Area are of high risks of high-temperature disasters^14^. With global warming, the population exposure to high temperature in North China where Xiongan New Area is located will increase significantly in the future^15^. The construction of Xiongan New Area will change local demographic pattern and attract more population. Therefore, the risk of high-temperature disasters in the future will be higher than the estimated results of previous study^15^. From the two indicators of annual average temperature and annual precipitation, it is found that the Haihe River Basin where Xiongan New Area is located experienced rising temperature and decreasing precipitation, and the climate becomes warmer and drier^12^. This study focused on the trend of climate change, and didn’t involve extreme changes in climate factors, while meteorological disaster risk research focuses more on the extremes of climate factors. Scholars from the National Climate Center used climate change scenario data to carry out a projection study of the extreme climate event index in Xiongan New Area and its adjacent areas^16,17^. These studies focused only on the extreme climate index. In a word, above-mentioned studies lack of systematic analysis of extreme values of key meteorological hazard factors. Extreme value analysis is of prime importance for natural disaster risk modeling and assessment. Most studies perform analysis of extreme values of meteorological factors under the assumption of stationary. An increasing number of studies are using non-stationary models to analyze extreme values of meteorological factors^18–20^. However, fewer studies have simulated extreme values under multiple climate scenarios at the grid scale. Moreover, fewer studies have carried out extreme value analysis of multiple meteorological factors from the perspective of natural disaster risk assessment. The extreme value analysis of multiple meteorological factors is more conductive to the comprehensive judgment of the hazard level of nature disaster. This paper aims to explore the future dynamics of extremes of multiple key meteorological hazard factors in Xiongan New Area using stationary and non-stationary GEV models. Annual maximum daily precipitation and annual maximum amount of consecutive precipitation are the key hazard factors for flood disaster^21–24^, annual maximum temperature and annual longest consecutive high-temperature days are the key hazard factors for high-temperature disaster^25,26^. From the perspective of meteorological disaster risk assessment, this paper explores the spatiotemporal dynamics of extremes for above-mentioned four key hazard factors under two climate scenarios during projection period at the grid scale. Now Xiongan New Area is under construction and it is of great practical importance to assess the spatiotemporal characteristics of extreme values of key meteorological hazard factors in Xiongan New Area for building a scientific and efficient disaster prevention system.

## Materials and methods

### Study area

**Figure 1:**
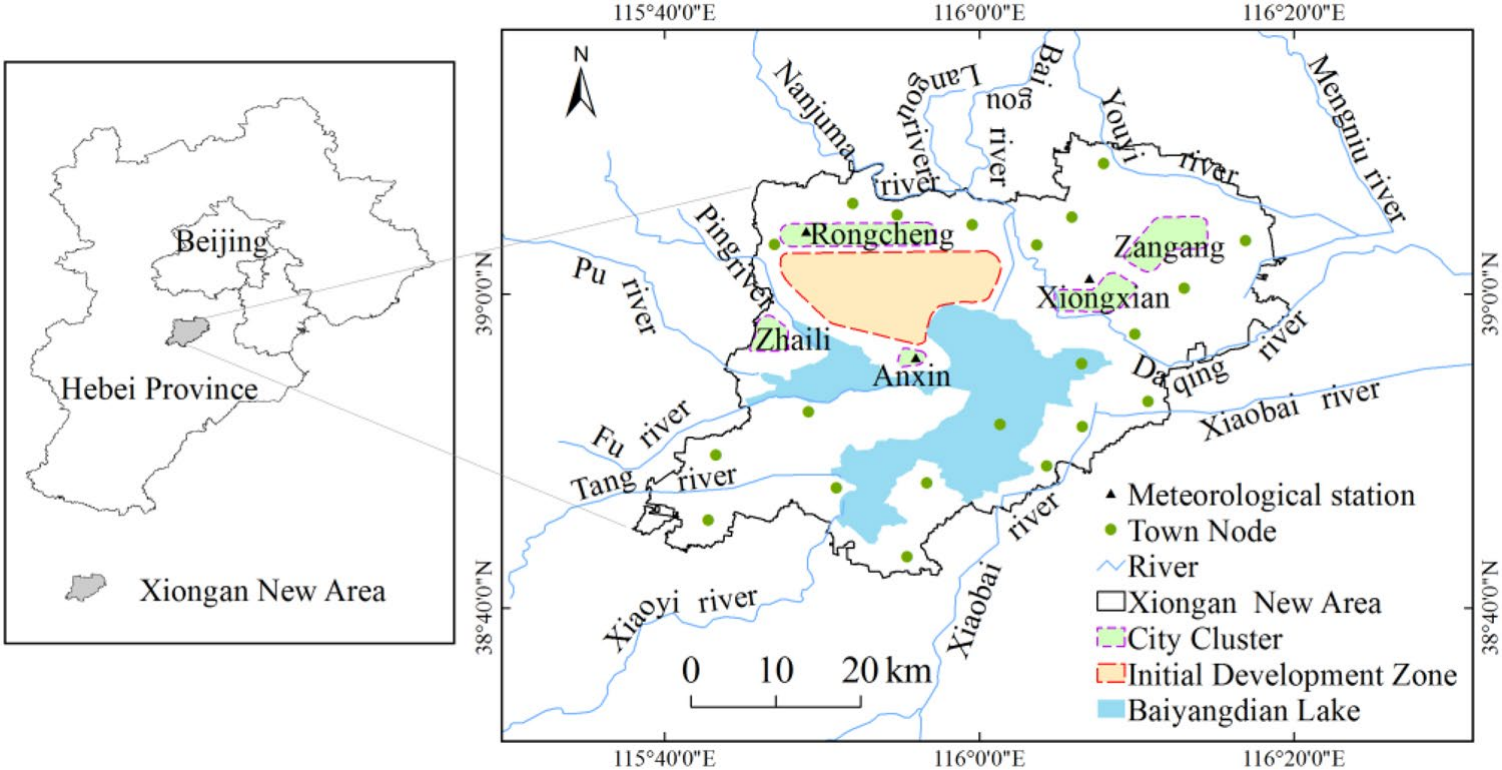
Location of Xiongan New Area and its urban–rural space layout. The map was generated using ArcGIS 10.1. Source: Map of Xiongan New Area comes from the master plan for Xiongan New Area.

Xiongan New Area will include the regions under the administrative jurisdiction of Xiongxian, Rongcheng and Anxin counties (including Baiyangdian Lake), Maozhou, Gougezhuang and Qijianfang townships of Renqiu city and Longhua township of Gaoyang county, with a planned area of 1,770 square kilometers^27^. Urban and rural space layout in Xiongan New Area includes one initial development zone, five city clusters and multiple town nodes (Fig. 1). Xiongan New Area locates in the east of Taihang Mountain and the south of Yanshan Mountain. It sits in the hinterland of the Daqing River of the Haihe River system. The upper reach of the Daqing River is subject to heavy rainstorms. The Zhulong River, Xiaoyi River, Tanghe River, Fuhe River, Caohe River, Pinghe River, Yangcun River, Puhe River and Baigou Diversion Channel all together flow into the Baiyangdian Lake. Xiongan New Area locates at a low-lying terrain, and has historically suffered from frequent floods.

### Data

The 6.25-km high-resolution and four-member downscaling dataset for future climate projections of daily precipitation amount and daily maximum temperature under RCP4.5 and RCP8.5 scenarios were used in this study. Both dynamical and statistical downscaling techniques were adopted to produce this dataset. Regional Climate Model Version 4.4^28^ was used to downscale four CMIP5 (Coupled Model Intercomparison Project Phase 5) global models, that is, CSIROMk3–6–0, EC-EARTH, HadGEM2-ES, MPI-ESM-MR, and then quantile mapping method was used to further downscale four regional climate simulations at the 6.25-km resolution^29,30^. The multi-model ensemble mean of the projection data can well reproduce the spatial distribution of most climate extremes^17^.

### Methods

Two key hazard factors, annual maximum daily precipitation and annual maximum amount of consecutive precipitation, were selected to study flood hazard, and two key hazard factors of annual maximum temperature and longest consecutive high-temperature days were selected to measure the hazard of high-temperature disaster. In this paper, high temperature refers to the weather with the daily maximum temperature of 35 °C or above^31^. Based on the climate projections of daily precipitation amount and daily maximum temperature under RCP4.5 and RCP8.5 scenarios, four key hazard factors with five return periods during the period 1991–2050 in each grid of each member of climate projection data were evaluated using GEV model. Four-member ensemble mean method was used to measure four key hazard factors with five return periods in each grid. GEV method was often used to simulate the distribution of precipitation extreme or temperature extreme^20,23,32–34^. The cumulative distribution function of the GEV can be expressed as^35,36^ :

1 $$G\left( {x,\mu ,\sigma ,\xi } \right) = \exp \left\{ { - \left( {1 + \xi \left( {\frac{x - \mu }{\sigma }} \right)} \right)^{{\frac{ - 1}{\xi }}} } \right\},\;\;\left( {1 + \xi \left( {\frac{x - \mu }{\sigma }} \right)} \right) > 0$$

where $\mu$ is the location parameter, $\sigma$ is the scale parameter and $\xi$ is shape parameter.

For a non-stationary process in this paper, the location parameter is assumed to be a linear fuction of time to account for non-stationary, while keeping the scale and shape paremeters constant:

2 $$\mu \left( t \right) = \mu_{1} t + \mu_{0}$$

where $t$ is the time (in years), $\mu_{0}$ is constant and $\mu_{1}$ is the slope of the time-variant parameter.

Time series of four hazard factors were checked for non-stationarity using two methods of Mann–Kendall trend analysis and linear trend analysis. If the results of Mann–Kendall trend analysis or linear trend analysis show that the time series in one grid is significant at the 5% level, the time series is non-stationary and its generalized extreme value varies between the minimum and the maximum of the generalized extreme value; Otherwise, the time series is stationary and its generalized extreme value is constant. Mann–Kendall trend was analyzed by CRAN-R package modifiedmk and linear trend was performed by function lm in R. Time series of four hazard factors were ‘pre-whitened’ to eliminate the effect of serial correlation before applying the Mann–Kendall test if the lag-1 serial correlation coefficient is significant at the 5% level^37^. Lag-1 serial correlation coefficient was calculated using function acf in R. Stationary GEV and time-varying GEV methods were executed by CRAN-R package extRemes (Version 2.1). Maximum likelihood estimation (MLE) was used to infer the GEV distribution parameters under stationary and non-stationary conditions and the “Nelder-Mead” algorithm was used for optimization. Initial estimates are calculated using L-moments estimates.

## Results and analysis

### Spatiotemporal dynamics of annual maximum daily precipitation and annual maximum amount of consecutive precipitation

During the period 1991–2050, annual maximum daily precipitation with five return periods in Xiongan New Area will increase by 2.0% to 3.6% under RCP4.5 scenario, but they will hardly change under RCP8.5 scenario (Table 1).

**Table 1:** Change of annual maximum daily precipitation with five return periods (once in T years) during the period 1991–2050 in Xiongan New Area based on climate projection data (unit: %).

Climate scenario	T = 10	T = 20	T = 30	T = 50	T = 100
RCP 4.5	3.6	3.0	2.7	2.4	2.0
RCP 8.5	0.1	0.0	0.0	0.0	0.0

**Figure 2:**
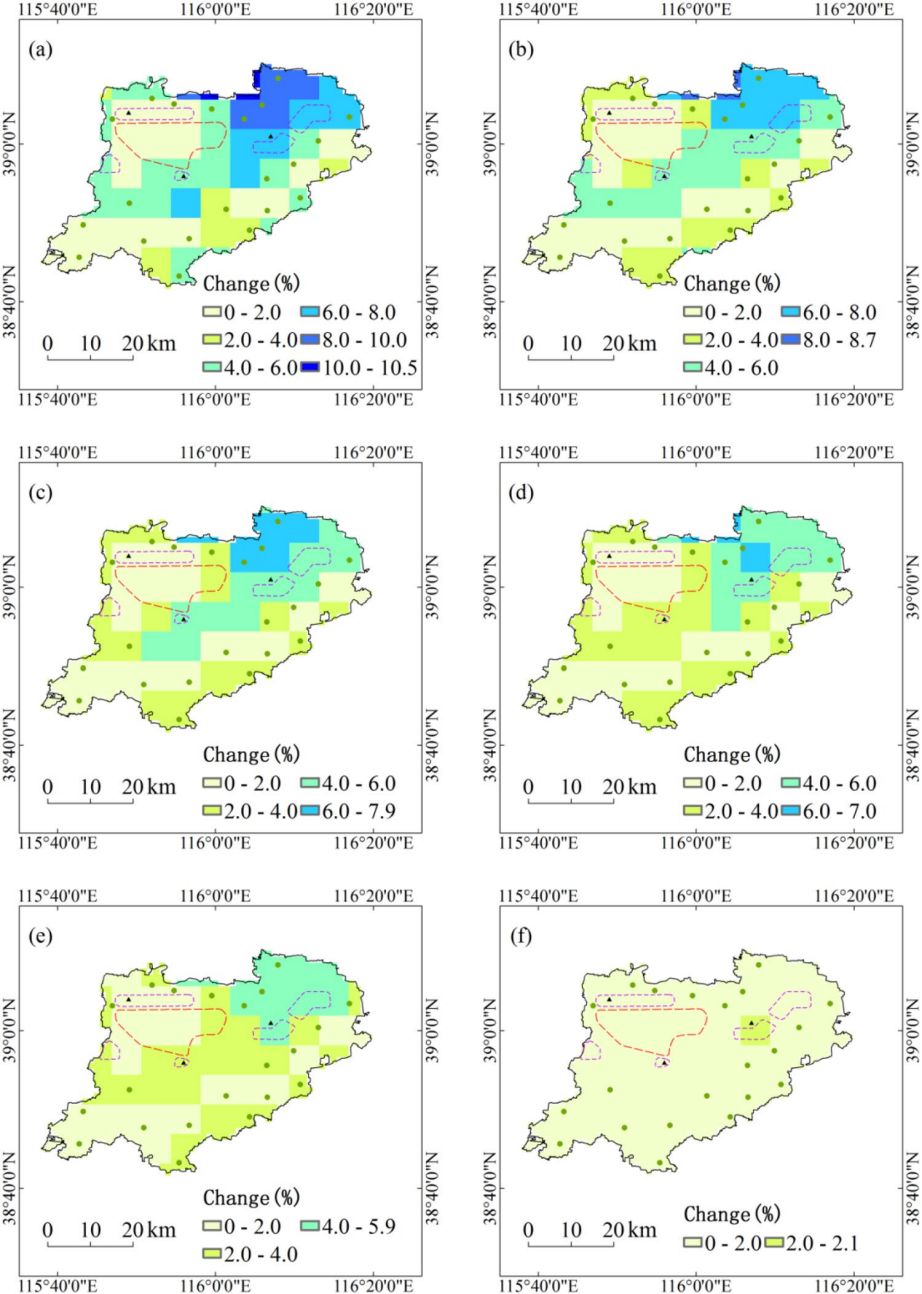

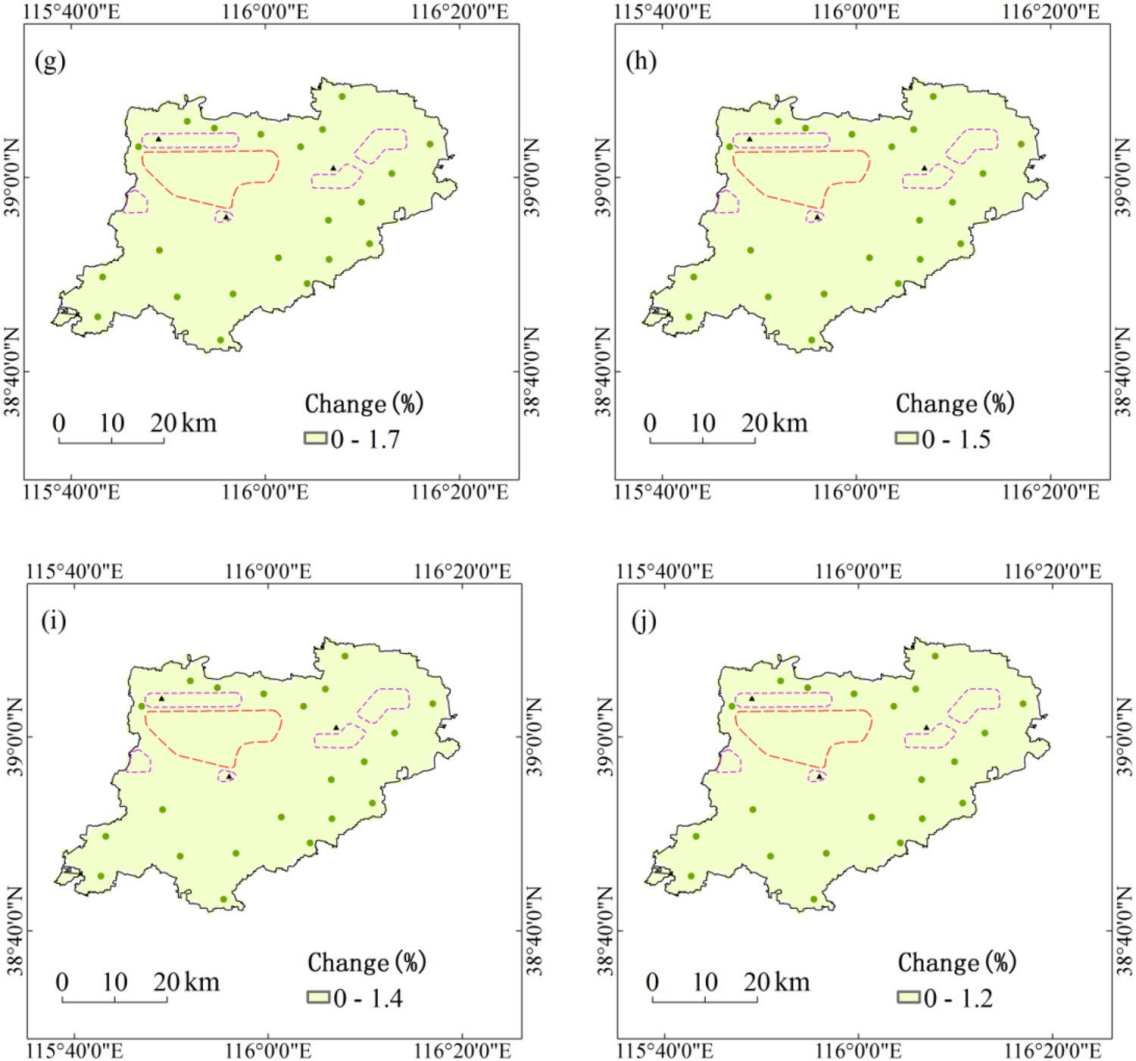
Projected changes of annual maximum daily precipitation with five return periods during the period 1991–2050. Maps show return periods of 10-year (**a**), 20-year (**b**), 30-year (**c**), 50-year (**d**) and 100-year (**e**) under RCP4.5 scenario and return periods of 10-year (**f**), 20-year (**g**), 30-year (**h**), 50-year (**i**) and 100-year (**j**) under RCP8.5 scenario. Maps generated in ArcGIS 10.1.

During the period 1991–2050, annual maximum daily precipitation with five return periods under RCP4.5 scenario in all regions of Xiongan New Area will increase by less than 10%, and the increase in the northeast of Xiongan New Area are relatively large. Therefore, two city clusters of Xiongxian and Zangang in the northeast should pay much attention to the increase of annual maximum daily precipitation with five return periods. With the increase of return period, the relative changes of extremes of annual maximum daily precipitation will generally decrease under RCP4.5 scenario. Under RCP8.5 scenario, annual maximum daily precipitation with five return periods in all regions of Xiongan New Area will increase by less than 2% (Fig. 2).

During the period 1991–2050, annual maximum amount of consecutive precipitation with five return periods in Xiongan New Area will only increase by 1.0% to 1.8% under RCP4.5 scenario and 0.7% to 1.3% under RCP8.5 scenario (Table 2).

**Table 2:** Change of annual maximum amount of consecutive precipitation with five return periods (once in T years) during the period 1991–2050 in Xiongan New Area based on climate projection data (unit: %).

Climate scenario	T = 10	T = 20	T = 30	T = 50	T = 100
RCP 4.5	1.8	1.5	1.3	1.2	1.0
RCP 8.5	1.3	1.1	1.0	0.8	0.7

**Figure 3:**
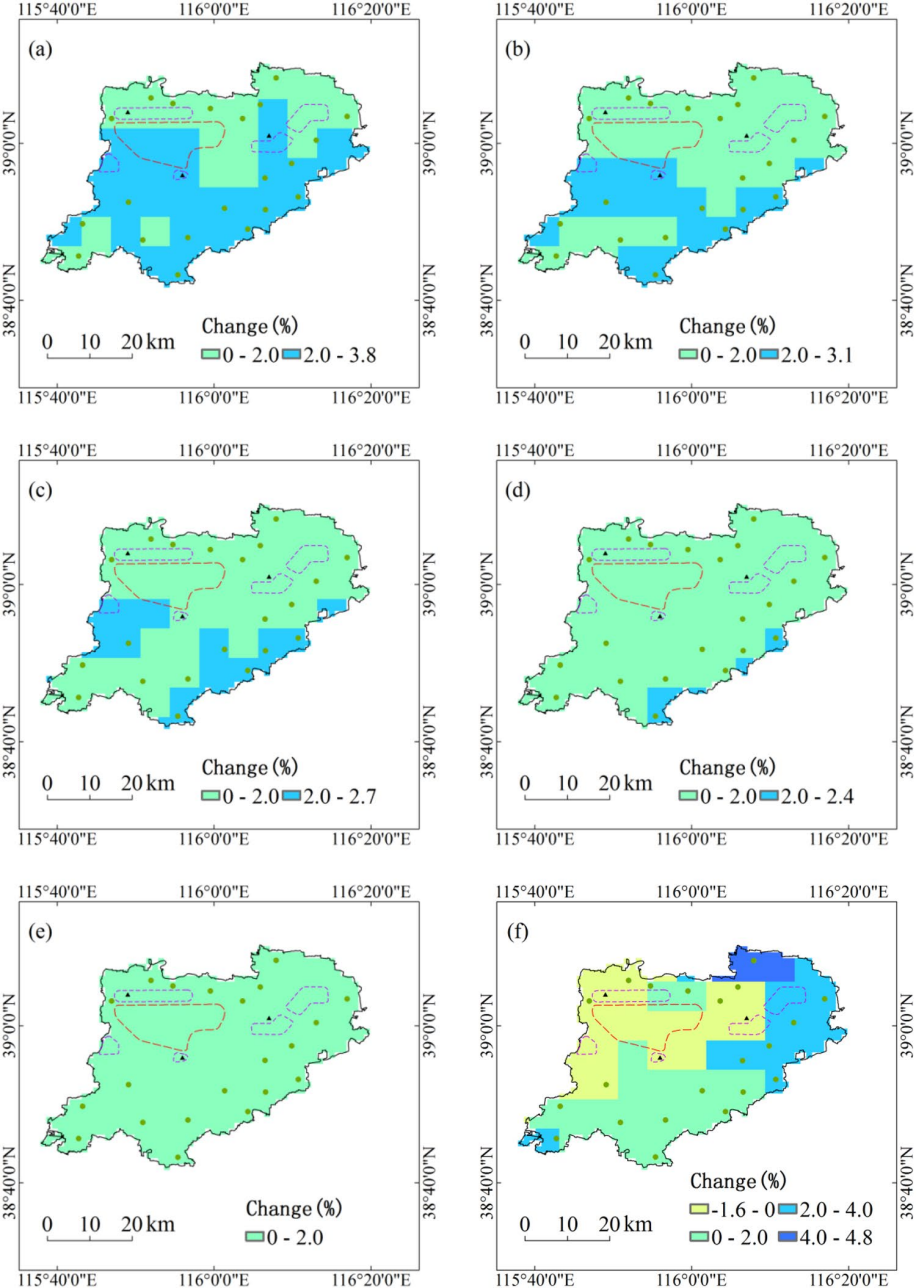

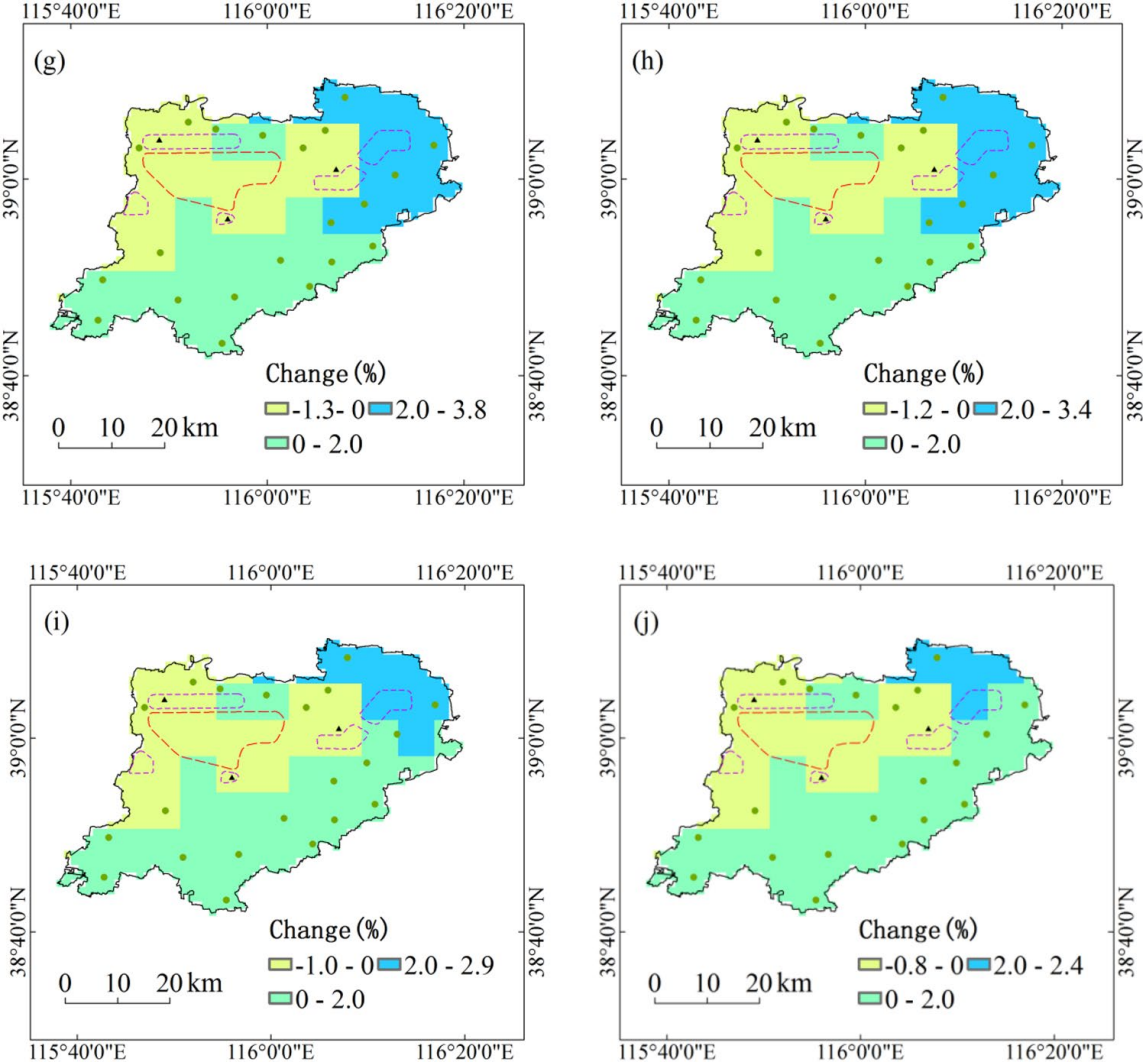
Projected changes of annual maximum amount of consecutive precipitation with five return periods during the period 1991–2050. Maps show return periods of 10-year (**a**), 20-year (**b**), 30-year (**c**), 50-year (**d**) and 100-year (**e**) under RCP4.5 scenario and return periods of 10-year (**f**), 20-year (**g**), 30-year (**h**), 50-year (**i**) and 100-year (**j**) under RCP8.5 scenario. Maps generated in ArcGIS 10.1.

During the period 1991–2050, annual maximum amount of consecutive precipitation with five return periods under RCP4.5 scenario in all regions of Xiongan New Area will increase by less than 4%, and the increase in initial development zone and five city clusters are relatively small. With the increase of return period, the relative changes of annual maximum amount of consecutive precipitation with five return periods will generally decrease under RCP4.5 scenario. Under RCP8.5 scenario, annual maximum amount of consecutive precipitation with five return periods will increase by 2% to 5% in the northeast of Xiongan New Area and 0 to 2% in the south, but decrease by less than 2% in the northwest (Fig. 3).

During the period 1991–2050, annual maximum daily precipitation with five return periods in the northeast of Xiongan New Area will increase relatively greatly under RCP4.5 scenario. Therefore, there will be relatively higher flood hazard in Xiongxian county and its adjacent region. But on the whole, flood hazard in Xiongan New Area will not increase too much.

### Spatiotemporal dynamics of annual maximum temperature and longest consecutive high-temperature days

During the period 1991–2050, annual maximum temperature with five return periods in Xiongan New Area will increase by 1.8 °C under RCP4.5 scenario and 2.3 °C under RCP8.5 scenario (Table 3).

**Table 3:** Change of annual maximum temperature with five return periods (once in T years) during the period 1991–2050 in Xiongan New Area based on climate projection data (unit: °C).

Climate scenario	T = 10	T = 20	T = 30	T = 50	T = 100
RCP 4.5	1.8	1.8	1.8	1.8	1.8
RCP 8.5	2.3	2.3	2.3	2.3	2.3

**Figure 4:**
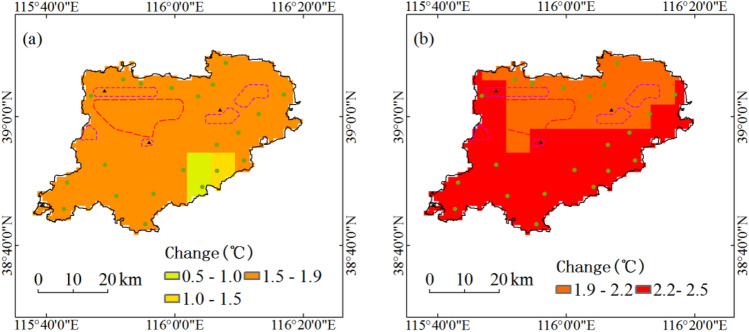
Projected changes of annual maximum temperature with five return periods during the period 1991–2050. Maps show return periods of 10-year, 20-year, 30-year, 50-year and 100-year under RCP4.5 scenario (**a**) and under RCP8.5 scenario (**b**). Maps generated in ArcGIS 10.1.

During the period 1991–2050, annual maximum temperature with five return periods under RCP4.5 scenario in most areas of Xiongan New Area will increase by above 1.5 °C (Fig. 4a). Under RCP8.5 scenario, annual maximum temperature with five return periods in Xiongan New Area will increase by above 1.9 °C, and the overall spatial distribution of the increase is greater in the south than that in the north (Fig. 4b).

During the period 1991–2050, annual longest consecutive high-temperature days with five return periods in Xiongan New Area will increase by 1.6d under RCP4.5 scenario and 2.5d under RCP8.5 scenario (Table 4).

**Table 4:** Change of annual longest consecutive high-temperature days with five return periods (once in T years) during the period 1991–2050 in Xiongan New Area based on climate projection data (unit:d).

Climate scenario	T = 10	T = 20	T = 30	T = 50	T = 100
RCP 4.5	1.6	1.6	1.6	1.6	1.6
RCP 8.5	2.5	2.5	2.5	2.5	2.5

**Figure 5:**
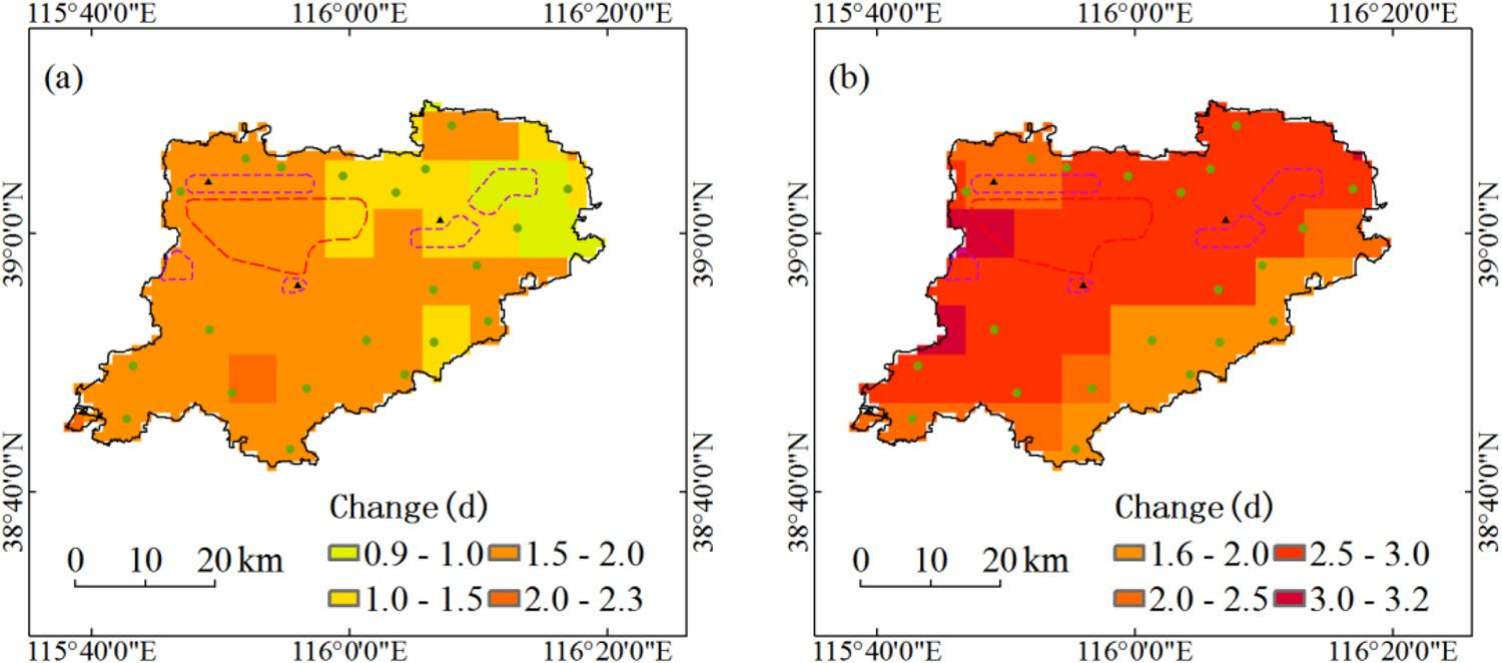
Projected changes of annual longest consecutive high-temperature days with five return periods during the period 1991–2050. Maps show return periods of 10-year, 20-year, 30-year, 50-year and 100-year under RCP4.5 scenario (**a**) and under RCP8.5 scenario (**b**). Maps generated in ArcGIS 10.1.

During the period 1991–2050, annual longest consecutive high-temperature days with five return periods under RCP4.5 scenario in the south and west of Xiongan New Area will increase by above 1.5d, and that in northeast will increase by 0.9 to 1.5d (Fig. 5a). Under RCP8.5 scenario, annual longest consecutive high-temperature days with five return periods in most areas of Xiongan New Area will increase by above 2.5d, and that in southeast will increase by 1.6 to 2.0d (Fig. 5b).

During the period 1991–2050, annual maximum temperature with five return periods and annual longest consecutive high-temperature days with five return periods under two scenarios of RCP4.5 and RCP8.5 will increase obviously. Therefore, there will be high hazard of high-temperature disaster in Xiongan New Area.

## Conclusions and recommendations

Based on 6.25 km high-resolution downscaling projection data, the spatiotemporal dynamics of key hazard factors are projected. The main conclusions are as follows:

1. At the spatial scale of the entire region, annual maximum daily precipitation and annual maximum amount of consecutive precipitation with five return periods under two scenarios of RCP4.5 and RCP8.5 during the period 1991–2050 in Xiongan New Area will not increase too much. During the period 1991–2050, annual maximum temperature with five return periods in Xiongan New Area will increase by 1.8 °C under RCP4.5 scenario and 2.3 °C under RCP8.5 scenario, and annual longest consecutive high-temperature days with five return periods in Xiongan New Area will increase by 1.6d under RCP4.5 scenario and 2.5d under RCP8.5 scenario.
2. At the grid scale, only in the northeast of Xiongan New Area and under RCP4.5 scenario will annual maximum daily precipitation with five return periods increase by 4% to 10%. Annual maximum daily precipitation with five return periods in most areas of Xiongan New Area under RCP4.5 scenario will increase by less than 4% and that in all regions of Xiongan New Area under RCP8.5 scenario will increase by less than 2%. Annual maximum temperature with five return periods will increase by above 1.5 °C under RCP4.5 scenario in most grids and above 1.9 °C under RCP8.5 scenario in all grids. Annual longest consecutive high-temperature days with five return periods under RCP4.5 scenario in the south and west of Xiongan New Area will increase by above 1.5d, and that in northeast will increase by 0.9 to 1.5d. Under RCP8.5 scenario, annual longest consecutive high-temperature days with five return periods in all grids of Xiongan New Area will increase by above 1.6d.
3. On the whole, the hazard of flood disaster will hardly show any change up to 2050, but there will be relatively higher flood hazard in Xiongxian county and its adjacent region. All regions of Xiongan New Area will face high hazard of high-temperature disaster.
